# NRF2 DLG Domain Mutations Identified in Japanese Liver Cancer Patients Affect the Transcriptional Activity in HCC Cell Lines

**DOI:** 10.3390/ijms22105296

**Published:** 2021-05-18

**Authors:** Effi Haque, Magdalena Śmiech, Kamila Łuczyńska, Marie France Bouchard, Robert Viger, Hidetoshi Kono, Mariusz Pierzchała, Hiroaki Taniguchi

**Affiliations:** 1 Laboratory for Genome Editing and Transcriptional Regulation, Institute of Genetics and Animal Biotechnology, Polish Academy of Sciences, 05-552 Jastrzębiec, Poland; e.haque@igbzpan.pl (E.H.); m.smiech@igbzpan.pl (M.Ś.); k.luczynska@igbzpan.pl (K.Ł.); 2 Reproduction, Mother and Child Health, Centre de Recherche du CHU de Québec-Université Laval and Centre de Recherche en Reproduction, Développement et Santé Intergénérationnelle (CRDSI), Quebec, QC GIV4G2, Canada; Marie-France.Bouchard@crchudequebec.ulaval.ca (M.F.B.); robert.viger@crchudequebec.ulaval.ca (R.V.); 3 Department of Obstetrics, Gynecology, and Reproduction, Université Laval, Quebec, QC G1V0A6, Canada; 4 Molecular Modeling and Simulation Group, National Institutes for Quantum and Radiological Science and Technology, Kizugawa, Kyoto 619-0215, Japan; kono.hidetoshi@qst.go.jp; 5 Department of Genomics and Biodiversity, Institute of Genetics and Animal Biotechnology, Polish Academy of Sciences, 05-552 Jastrzębiec, Poland; m.pierzchala@igbzpan.pl

**Keywords:** NRF2, KEAP1, somatic mutation, transcriptional activity, BRAF, MMP9, HCC

## Abstract

Geographically, East Asia had the highest liver cancer burden in 2017. Besides this, liver cancer-related deaths were high in Japan, accounting for 3.90% of total deaths. The development of liver cancer is influenced by several factors, and genetic alteration is one of the critical factors among them. Therefore, the detailed mechanism driving the oncogenic transformation of liver cells needs to be elucidated. Recently, many researchers have focused on investigating the liver cancer genome and identified somatic mutations (MTs) of several transcription factors. In this line, next-generation sequencing of the cancer genome identified that oxidative stress-related transcription factor NRF2 (NFE2L2) is mutated in different cancers, including hepatocellular carcinoma (HCC). Here, we demonstrated that NRF2 DLG motif mutations (NRF2 D29A and L30F), found in Japanese liver cancer patients, upregulate the transcriptional activity of NRF2 in HCC cell lines. Moreover, the transcriptional activity of NRF2 mutations is not suppressed by KEAP1, presumably because NRF2 MTs disturb proper NRF2-KEAP1 binding and block KEAP1-mediated degradation of NRF2. Additionally, we showed that both MTs upregulate the transcriptional activity of NRF2 on the *MMP9* promoter in Hepa1-6 and Huh7 cells, suggesting that MT derived gain-of-function of NRF2 may be important for liver tumor progression. We also found that ectopic overexpression of oncogenic BRAF WT and V600E increases the transcriptional activity of NRF2 WT on both the 3xARE reporter and *MMP9* promoter. Interestingly, NRF2 D29A and L30F MTs with oncogenic BRAF V600E MT synergistically upregulate the transcription activity of NRF2 on the 3xARE reporter and *MMP9* promoter in Hepa1-6 and Huh7 cells. In summary, our findings suggest that MTs in NRF2 have pathogenic effects, and that NRF2 MTs together with oncogenic BRAF V600E MT synergistically cause more aberrant transcriptional activity. The high activity of NRF2 MTs in HCC with BRAF MT warrants further exploration of the potential diagnostic, prognostic, and therapeutic utility of this pathway in HCC.

## 1. Introduction

More than 50% of the global liver cancer burden is located in East Asia. After China (51.03%), liver cancer-related deaths are highest in India and Japan, accounting for 4.33% and 3.90%, respectively, of the global deaths in 2017 [1]. Epidemiologically, alcohol consumption and hepatitis virus (HBV, HCV) infection, as well as the occurrence of nonalcoholic fatty liver disease (NAFLD) and nonalcoholic steatohepatitis (NASH), have been reported as risk factors for hepatocellular carcinoma (HCC) [2,3,4,5,6,7,8]. Furthermore, the primary etiological factor for liver cancer in Japan is HCV infection [1]. HCV infection causes oxidative stress and activates nuclear factor erythroid-2–related factor 2 (NRF2) [9]. NRF2 is an oxidative stress-related transcription factor reported as a potential prognostic marker for HCC development and progression [10,11]. However, the detailed understanding of how NRF2 reacts as oncogene in liver cells remains unknown. Recent findings suggest that NRF2 promotes cancers because of somatic mutations (MTs) that cause aberrant NRF2 transcriptional activity [12]. Whole exome sequence analysis identified that around 6.4% of MTs found in tissues of HCC-affected patients are present in the NRF2 gene. Interestingly, these MTs are located within the *Asp*-Leu-Gly (DLG) and *Glu*-*Thr*-*Gly*-*Glu* (ETGE) motifs (KEAP1 binding elements) of NRF2, which are important for binding with its negative regulator, KEAP1 [13]. A search of the mutation database revealed that somatic MTs encompassing the NRF2-DLG motif cover a greater region than MTs in the ETGE motif [14]. Moreover, NRF2 MTs are an early event in rats fed with choline-devoid, methionine-deficient (CMD) diet-promoted preneoplastic hepatic nodules, and all MTs are confined within the DLG (74%) or ETGE (26%) motif of exon 2 of the NRF2 gene [15]. A study of diethylnitrosamine (DEN) induced HCC in mice revealed that MTs in the DLG motif of NRF2 are a crucial driver for HCC [10]. Besides these, it was reported that V32E represents the most frequent DLG MT (weak bond), while T80A is the most frequent ETGE MT (strong bond) [14]. This unique nature of KEAP1 binding with DLG motif enables the prompt response of NRF2 to oxidative and electrophilic stress [14]. MTs in this domain specifically alter the amino acids that affect the interaction between NRF2 and KEAP1. Moreover, it has been reported that the DLG motif is a weaker KEAP1-binding site than the ETGE motif. This makes the DLG region more vulnerable to structural changes, and any MTs in this motif are predicted to have a great influence on tumor growth [14,16]; however, the functional importance of these DLG MTs in liver cancer cells is not well understood. Several lines of evidence indicate that overexpression of NRF2 is highly related to cancer development [10,11,15]. The KO of Nrf2 using mice suggested that suppression of aberrant NRF2 activity could reduce the tumor burden [10]. Interestingly, loss of function MTs in KEAP1 overactivate NRF2 and provide benefits for lung cancer cell growth [17]. It is possible that NRF2 might interact with other signaling pathways, which control the tumor survival signal as a result of NRF2 overactivation.

A recent study reported that the matrix metalloproteinase (MMP) family gene MMP9 is one of the targets of NRF2; the MMP9 gene contains two putative antioxidant response elements (ARE), which are known target sequences for NRF2, in its promoter region [18]. Interestingly, NRF2 promotes invasion in human HCC partly through regulating the expression of MMP9 [11]. An in vitro study with HepG2 cells showed that upregulation of the NRF2 pathway stimulates target gene expression, including MMP9, which increases the invasiveness of HCC cells [11]. On the other hand, BRAF (v-raf murine sarcoma viral oncogene homolog B1) is described as a potential oncogene that plays an important role in NRF2 activation [19]. It has been reported that BRAF V600E MT is responsible for melanoma progression through activation of the downstream MEK/ERK pathway [20]. BRAF phosphorylates ERK via MEK in cancer cells, and phospho-ERK phosphorylates its downstream targets, which include NRF2 [21,22,23]. During tumorigenesis, oncogenic BRAF has been reported to augment NRF2 activity [21]. Cancer cells with NRF2 MTs exhibit high levels of transcriptional activity and maintain malignant tumor growth [24]. Moreover, higher levels of MMP-9 and BRAF V600E MT are associated with lower progression-free survival and overall survival [25]. However, no conclusive findings on the occurrence and transcriptional activity of oncogenic MTs in the coding region regulating the tumor progression process have yet been published. On the basis of these observations, we hypothesized that MTs in the coding region of NRF2 might cause aberrant transcriptional activity and have some effect on MMP9 transactivation when BRAF MT is also present in liver cancer cells. 

Our study successfully revealed that NRF2 gene MTs found in HCC increase the transcriptional activity of NRF2. MTs cause NRF2 to lose its normal structure and hamper a NRF2-KEAP1 interaction. We also found that NRF2 MTs induce the transcriptional activity of the *MMP9* promoter, thereby driving increased *MMP9* expression that is linked to tumor invasion [11,26]. Furthermore, NRF2 D29A and L30F MTs together with BRAF V600E MT play crucial roles in hepatic transcriptional regulation. 

## 2. Results

### 2.1. NRF2 Mutations Are Mostly Located in the DLG Motif of NRF2

**Figure 1:**
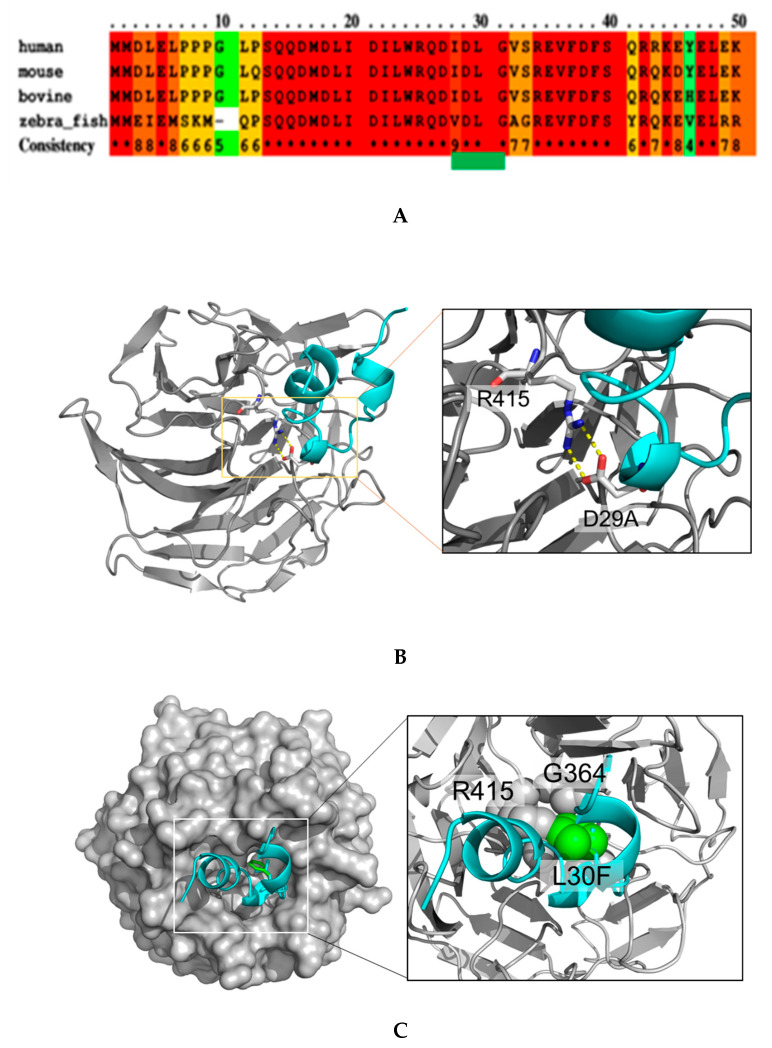
Evolutionally conserved NRF2 DLG domain and structural simulation of KEAP1/NRF2 MTs complex. (**A**) The alignment of the human, mouse, bovine, and zebrafish Nrf2 amino acid sequence. The red color box shows highly conserved (100%) elements among the species. DLG domain is indicated by green lines. (**B**) KEAP1 (gray) and NRF2 DLG (cyan) are shown by cartoon model. Hydrogen bonds between R415 of KEAP1 and D29 of NRF2 are shown by yellow dotted lines. (**C**) KEAP1 and NRF2 DLG are represented by surface (colored in gray) and cartoon (colored in cyan) models, respectively. In the enlarged view, G364 and R415 of KEAP1 are represented by gray spheres, whereas L30F of NRF2 is represented by a green sphere. All images were drawn using PyMOL.

To evaluate the prevalence of NRF2 MTs in different human cancers, we searched the International Cancer Genome Consortium (ICGC) database of different cancers and found that NRF2 somatic MTs in different cancers were mostly located at DLG motifs of NRF2 (Table 1) [27]. The whole-genome sequencing analysis of Japanese liver cancer patients identified two somatic MTs found in DLG domains. From an evolutionary perspective, MTs in the DLG motif found in this study are well conserved among various species (Figure 1A). The highly conserved elements among the analyzed species (human, mouse, bovine, and zebrafish) are highlighted in red and are 100% conserved throughout the different species and the highly conserved DLG domain is indicated by green lines. The MTs in such evolutionary conserved elements suggest a strong effect on protein structure and function. Since D29 and L30 are located in the functional domain of NRF2 [14], it is expected that MTs at these sites have a significant impact on DNA-binding and protein stability. To examine the effect of the MTs from a structural point of view, we modelled the mutants based on a crystal structure (PDB code: 3wn7) [14] using PyMOL Molecular Graphics System, Version 2.0 Schrödinger, LLC. For the D29A MT, NRF2 loses two hydrogen bonds with R415, reducing the binding affinity by at least 4 kcal/mol. In addition, D29A produces a cavity in the binding site, causing further reductions in binding affinity (Figure 1A,B). NRF2 with a L30F MT will not be able to fit into the pocket at the KEAP1 surface because the MT causes a structural clash between L30F of NRF2 and R415 and G364 of Keap1 (Figure 1C). Thus, these data suggest that NRF2 MTs in these regions may trigger abberant NRF2 transcriptional activity and impact liver carcinogenesis. However, the functional importance of these MTs in HCC remains to be studied. 

### 2.2. NRF2 DLG Mutations Have a Gain-of-Function Activity

**Figure 2:**
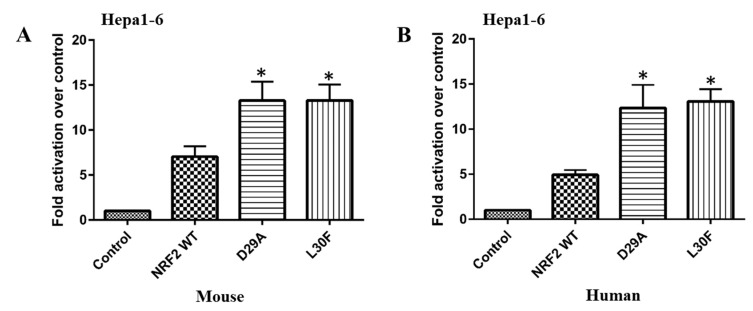
NRF2 MTs increase transactivation potential on its target promoter regions. (**A**). The transcriptional activity of mouse NRF2 WT and MTs. (**B**). The transcriptional activity of the human NRF2 WT and MTs in Hepa1-6 cells. In both experiments, cells were cotransfected with 3xARE luciferase reporters along with either an empty expression vector (serving as a control) or expression vectors (50 ng) for the indicated NRF2 in 24-well culture plates. The bars indicate fold activation of NRF2 WT and MTs (vs. control) on a NRF2 target promoter. Promoter activity is reported as fold activation over control. Data represent the mean ± SEM of 9 (**A**) and 5 (**B**) independent experiments (*, *p* < 0.05).

In our study, mouse Hepa1-6 cells were used to test whether NRF2 MTs have aberrant transcriptional activity in HCC. The possibility was explored by transfecting Hepa1-6 cells with mouse WT NRF2 or NRF2 MTs (D29A and L30F) in the presence of a 3xARE reporter. Luciferase reporter assay showed that the transcriptional activity of NRF2 D29A and L30F MTs was increased compared to NRF2 WT (Figure 2A). Additionally, we compared the transcriptional activities of human NRF2 MTs (D29A and L30F) to that of human WT NRF2. We found that although human NRF2 WT can increase ARE-luciferase activity, D29A and L30F MTs were more than two-fold active when compared to the WT (*p* < 0.05). Together these data indicate that NRF2 MTs are associated with gain-of-function activity (Figure 2B). This suggests that the DLG domain is important to maintain proper NRF2 transcriptional activity, and MTs in this domain disrupt proper transcriptional regulation, which can lead to HCC development by increasing the activity of several cancer-related genes.

### 2.3. KEAP1 Expression Fails to Reduce the Transcriptional Activity of NRF2 MTs

**Figure 3:**
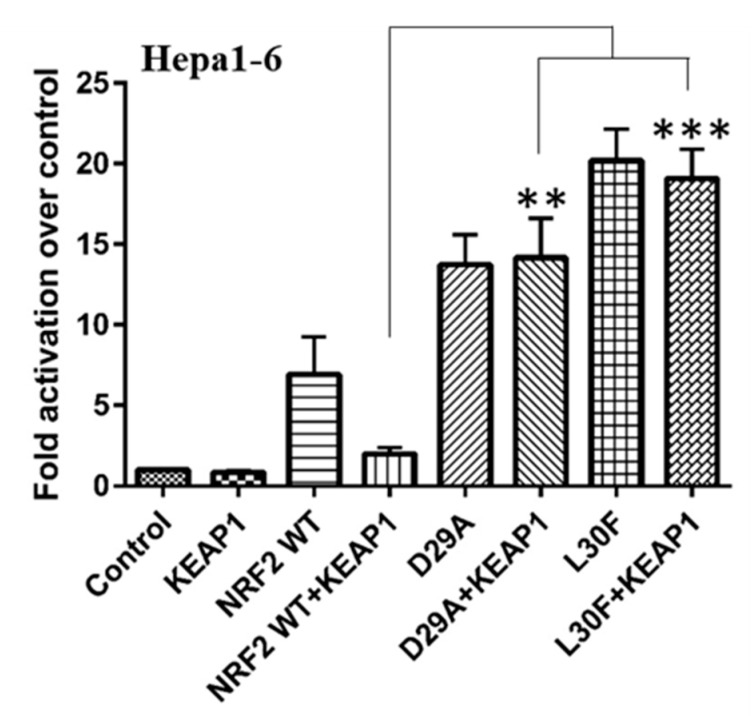
The effect of KEAP1 on NRF2 WT and MTs transcriptional activity in Hepa1-6 cells. The co-expression of KEAP1 inhibited the transcription activity of NRF2 WT but not of the D29A and L30F MTs, indicating that MTs block KEAP1-mediated regulation. The bars indicate fold activation of NRF2 WT and MTs (vs. control) on a NRF2 target promoter. The data represent the mean ± SEM of 4 independent experiments (**, *p* < 0.01; ***, *p* < 0.001).

Given the importance of the NRF2-KEAP1 system in cancer, we evaluated the effect of KEAP1 on NRF2 DLG MTs in HCC. The D29A and L30F base substitutions might affect the DLG motif within the Neh2 domain by altering the sequence to ALG/DFG. A defective interaction among KEAP1–NRF2 would then result in NRF2 accumulation and thus increased expression of NRF2 transcriptional targets [12]. Because KEAP1 is a negative regulator of NRF2, we proceeded to analyze the transcriptional activity of NRF2 MTs in the presence of KEAP1. The transcriptional activity of NRF2 was determined by analyzing the activity of 3xARE-luciferase reporter plasmids. NRF2 WT and both D29A and L30F MTs were overexpressed in Hepa1-6 cells in the presence or absence of HA-tagged KEAP1. D29A and L30F NRF2 MT proteins had higher transcriptional activity than NRF2 WT (Figure 3). As expected, the transcriptional activity of NRF2 WT was markedly decreased with KEAP1 co-expression. Interestingly, the presence of KEAP1 did not inhibit the transcriptional activity of NRF2 D29A and L30F MTs (Figure 3). This suggests that loss of KEAP1 function occurs when NRF2 D29A and L30F are mutated, and this translates to increased NRF2 transcriptional activity in HCC. 

### 2.4. NRF2 Mutations Increase the Transcriptional Activity of the MMP9 Promoter

**Figure 4:**
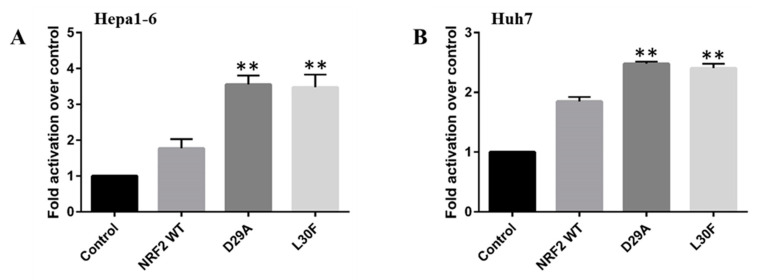
The ability of the NRF2 WT and MTs to transactivate *MMP9* promoters in Hepa1-6 (**A**) and Huh7 (**B**) cells. Cells were cotransfected with a *MMP9* luciferase reporter (250 ng) along with either an empty expression vector (serving as a control) or expression vectors (50 ng) for the indicated NRF2 in 24-well culture plates. Data represent the mean ± SEM of 6 (**A**) and 3 (**B**) independent experiments (**, *p* < 0.01).

Previous studies demonstrated that NRF2 levels correlate with invasiveness and metastatic progress of HCC through modulation of NRF2 expression [11]. Interestingly, NRF2 regulates the expression of MMP9, a protein regulating cell invasion in different cancers, including human HCC [11,18,28]. Therefore, we hypothesized that NRF2 MTs increase MMP9 transcription. To test this, we transiently cotransfected Hepa1-6 and Huh7 cells with the MMP9 promoter and human WT and MTs (D29A and L30F) NRF2. As predicted, NRF2 WT overexpression increased MMP9 promoter activity in both Hepa1-6 and Huh7 cells, which indicates that MMP9 expression is regulated by NRF2 in HCC. Interestingly, overexpression of NRF2 D29A and L30F MTs resulted in a significant enhancement of MMP9 promoter activity compared to NRF2 WT both in Hepa1-6 and Huh7 cells (Figure 4A,B). Taken together, these results suggest that NRF2 MTs increase MMP9 promoter activity in HCC cells, which might contribute to the invasiveness of liver cancer.

### 2.5. Both NRF2 and BRAF Mutations Increase the Transcriptional Activity of Target Promoters

**Figure 5:**
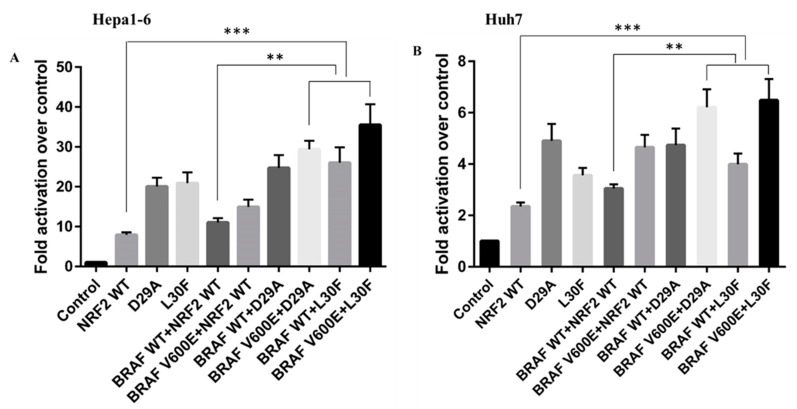
(**A**) The ability of NRF2 WT and MT expression plasmids together with oncogenic BRAF WT and V600E MT expression plasmids to transactivate a NRF2 target promoter (3xARE) in Hepa1-6 cells. (**B**) The ability of the NRF2 WT and MT expression plasmids together with oncogenic BRAF WT and V600E MT expression plasmids to transactivate the *MMP9* promoter when overexpressed in Huh7 cells. Data represent the mean ± SEM of 4 independent experiments (**, *p* < 0.01; ***, *p* < 0.001).

*BRAF* is one of the most described potential oncogenes. During tumorigenesis, oncogenic *BRAF* MT leads to activation of NRF2 [21]. Indeed, the presence of two oncogenic G12D K-Ras and V619E B-Raf MTs in murine primary cells increases the expression of NRF2, thereby inducing proliferation and tumorigenesis [21]. Our study examined the synergistic effect of NRF2 and BRAF MTs when overexpressed together, as NRF2 is the downstream target of BRAF-ERK [21]. To determine the synergistic role of NRF2 MTs together with oncogenic BRAF in HCC development, we overexpressed a 3xARE luciferase construct (which is sensitive to NRF2-mediated transactivation) along with NRF2 WT and MTs (D29A and L30F) and BRAF WT and BRAF V600E MT in Hepa1-6 cells. NRF2 D29A and L30F MTs showed higher transcriptional activity compared to WT NRF2 (Figure 5A). Likewise, when NRF2 MTs were overexpressed with BRAF WT, it showed higher transcriptional activity compared to NRF2 MTs alone (Figure 5A). Furthermore, overexpression of BRAF V600E MT yielded higher levels of NRF2 transcriptional activity compared to BRAF WT. Interestingly, there was a marked increase in NRF2 transcriptional activity when both NRF2 (D29A and L30F) and BRAF V600E MTs were overexpressed (Figure 5A), suggesting that NRF2 MTs achieve more gain-of-function activity when there is another oncogenic MT present.

Many lines of evidence suggest that BRAF MT is associated with the upregulation of MMP9 expression in several cancers [29,30]. Next, we tested whether BRAF MTs could regulate NRF2 transactivation activity by regulating MMP9 promoter activity in Huh7 cells. We therefore transfected Huh7 cells with a MMP9 promoter reporter in the presence of NRF2 WT or MTs (D29A and L30F) with or without BRAF WT or BRAF V600E. We found that NRF2 MTs overexpressed with BRAF WT showed synergistic induction of MMP9 promoter activity compared to NRF2 MTs alone (Figure 5B). BRAF V600E MT together with NRF2 (D29A, L30F) MTs resulted in an even higher level of NRF2 transcriptional activity as assessed through the induction of MMP9 promoter activity. MMP9 levels are increased in BRAF V600E expressing tumors [25]. Therefore it was not surprising that MMP9 promoter activity in the presence of NRF2 (D29A and L30F) MTs was found to be lower when BRAF WT was added to the mix when compared to both BRAF MTs together (Figure 5B). Taken together, these data suggested that BRAF MT is synergistically involved with NRF2 MTs in the upregulation of NRF2 transcriptional activity through increased MMP9 transcription. 

## 3. Discussion

Many previous studies have shown that MTs in NRF2 play a role in cancer progression [31,32,33]. We summarize NRF2 MTs in different cancers and MTs in NRF2 involved with the overactivation of NRF2. The D29A and L30F MTs are found in the DLG motif of the Neh2 domain of NRF2, and this domain is essential for ubiquitination and degradation of NRF2 [34,35]. It has been reported that the structure of the DLG motif is crucial for maintaining proper NRF2 turnover and NRF2-ARE mediated gene expression. Thus, any genetic alteration of *NRF2* might affect its transcriptional activity [36]. Moreover, MTs in the DLG motif change the conserved D29 and L30 residues, which makes it more vulnerable to structural changes [12]. Interestingly, our structural model of NRF2-KEAP1 indicates that the D29A MT in NRF2 drastically reduces its binding affinity with KEAP1, and the L30F MT causes structural hindrance to the pocket in the interface of NRF2 and KEAP1, also resulting in a decrease in binding affinity. Therefore, it is possible that MTs in functional domains of NRF2 may increase the risk of liver cancer by changing their proper structure and function. To this end, our study focused on DLG motif MTs found in several cancers, including HCC [10,37,38]. Previous studies revealed that 6.4% of *NRF2* MTs occur in HCC patients, and constitutive activation of NRF2 occurs more frequently in HCC cases [13,32,37,39,40,41]. In our study, we observed a constant increase in NRF2 D29A and L30F MT transcriptional activity through antioxidant response element (3xARE)-dependent luciferase reporter gene upregulation. Our findings are in agreement with a previous report of a DEN-induced HCC mouse model that developed DLG MTs in amino acid residues at the position of 29 (80%) and 32 (100%). MTs in those positions were associated with NRF2 overactivation [10]. All the genetic alterations in NRF2 DLG may share a common scenario where all MTs result in the overactivation of NRF2. However, further studies to test the transcriptional activity of all NRF2 DLG MTs are needed to clarify this point. 

Under homeostatic conditions, NRF2 is maintained at a very low intracellular concentration through its association with KEAP1 and the Cul3 E3 ligase [42]. Thus, any changes in the DLG motif are vulnerable to KEAP1-dependent polyubiquitination [34]. Moreover, this results in the constitutive activation of NRF2. It has been reported that elevated expression of NRF2 target genes confers advantages in terms of stress resistance and cell proliferation in normal and cancer cells [17]. In our study, we found that KEAP1 co-expression in Hepa1-6 cells visibly reduces the transcriptional activity of NRF2 WT; however, KEAP1 could not reduce the transcriptional activity of NRF2 D29A and L30F MT. Moreover, it has been demonstrated that NRF2 L30F is reductant to KEAP1 mediated protein degradation [12]. These results suggest that NRF2 MTs lead to aberrant transcriptional activity in HCC and induce tumor progression in HCC via upregulation of several NRF2 target genes. In agreement with this, several reports have indicated that both NRF2 DLG and KEAP1 MT can upregulate NRF2 transcriptional activity [12,43]. 

MMP-9 is important for invasion, metastasis, and tumor angiogenesis [44], and its expression is known to be upregulated in several cancer cells, including HCC [11,44]. The invasion process of MMP9 occurs through its positive correlation with NRF2 and high NRF2 expression in HCC patients associated with a poor prognosis [11]. It has been reported that transcriptional activation of *MMP9* is regulated by NRF2 [18]. It is possible that these phenomena strongly affect the development of malignant phenotypes. Our results suggest that *NRF2* MTs trigger tumor development. The results from our study showed that NRF2 D29A and L30F MTs regulate the transcriptional activity of the *MMP9* promoter through NRF2 induction in Hepa1-6 and Huh7 cells, which suggests that MTs are linked with the development of HCC. Notably, D29 and L30 are the most frequent *NRF2* gene MTs identified in different human tumors [12,31,32]. However, both D29A and L30F MTs are poorly represented in HCC metastases. We can speculate that overactivation of NRF2 caused by DLG domain MTs in Hepa1-6 and Huh7 cells leads to overexpression of *MMP9* that enhances tumor cell invasion and metastasis. Therefore, aberrant NRF2 expression that increases *MMP9* promoter activity in HCC cells can be considered as a critical target for the development of novel therapeutics.

Oncogenic signaling pathways, including oncogenic *B-RAF* (V619E), have been reported to augment NRF2 transcription via activation of the B-Raf-MEK-ERK and support its pro-tumorigenic effects [21]. Moreover, the activation of BRAF stimulates the transcription of NRF2 via activation of JUN and MYC [21]. In line with this speculation, our study for the first time, provides evidence that the transcriptional activity of NRF2 D29A and L30F MTs were increased with the overexpression of BRAF V600E MT. Recently, one group showed that BRAF MTs altered the tumor microenvironment by regulating the MAPK pathway, and MAPK activation is involved in NRF2 nuclear translocation [45]. Moreover, the overexpression of MAPK pathways is linked with the overexpression of ERK, which leads to the overexpression of several genes involved in tumor development, including *MMP9* [25,45]. Importantly, high levels of MMP9 and BRAF V600E MTs are associated with poor progression-free survival in melanoma patients [25], and activation of NRF2 through this pathway might be critical for tumor cell proliferation. In our study, we have shown a novel molecular mechanism by which BRAF and NRF2 MTs positively regulate transactivation of the *MMP9* promoter in Huh7 cells through NRF2 induction. This mechanism might contribute to HCC cell invasion and metastasis. 

**Figure 6:**
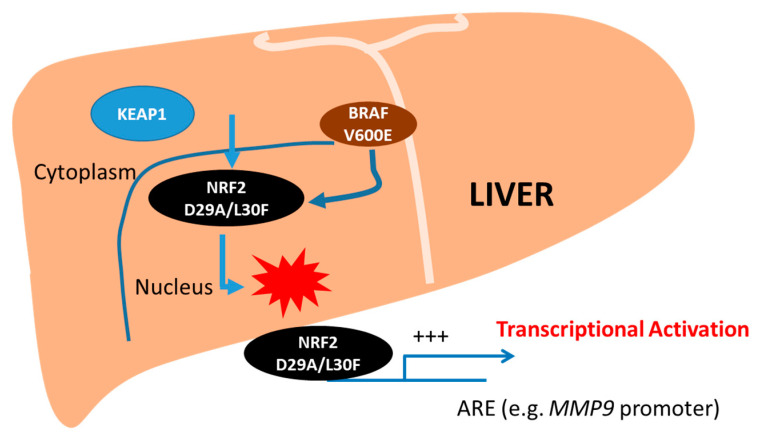
Graphical representation of mutant NRF2 transcriptional activity on target promoters. Novel human NRF2 MTs (D29A, L30F) disturb proper binding to KEAP1 and go to the nucleus, leading to an increase in transcriptional activity. BRAF V600E MT induces NRF2 MT transcriptional activity through increased MMP9 transcription. The increased transcriptional activity caused by NRF2-BRAF-MMP9 signaling may induce cell proliferation and invasion in liver tumors.

As summarized in Figure 6, our results have revealed a critical role played by a NRF2-BRAF-MMP9 signal that could serve as a basis for HCC progression when genes are dysregulated. Our findings could also suggest how MTs in cytoprotective genes can cause aberrant transcriptional activity in a synergistic manner that could lead to the activation of several genes responsible for creating a tumor phenotype. However, the high activity of NRF2 MTs in HCC with BRAF MT warrants further exploration of the potential diagnostic, prognostic, and therapeutic utility of this pathway in HCC.

## 4. Materials and Methods

### 4.1. Cell Culture

Hepa1-6 mouse hepatoma cells (Hepa1-6 cells) and human hepatocyte-derived carcinoma cells (Huh7 cells) were cultured in Dulbecco’s modified Eagle medium (DMEM) supplemented with 4.5 g/L of glucose (Lonza, Basel, Switzerland), 10% fetal bovine serum (FBS) (EURx, Gdansk, Poland), 100 units/mL of penicillin, and 100 units/mL of streptomycin (Lonza, Basel, Switzerland). Cells were maintained under standard conditions: 5% CO_2_, temp. 37 °C, humidified atmosphere in the Heracell 150i (Thermo Fisher Scientific, Waltham, MA, USA) incubator. Briefly, the Hepa1-6 and Huh7 cells (2 × 10^4^ cells) were cultured in 24-well plates in DMEM containing 10% FBS and 1% penicillin-streptomycin (Lonza, Basel, Switzerland).

### 4.2. Plasmids and Primers

Human NRF2 expression plasmid constructs carrying modifications of the WT gene in this study have been published by others and made available through Addgene or from other researchers. These plasmids were human NRF2 WT plasmid (NC16 pCDNA3.1 FLAG NRF2), pcDNA3-HA-KEAP1 (from Dr. Masayuki Yamamoto). Mutant variants of human and mouse NRF2 D29A and L30F were created through site-directed mutagenesis by using a site-directed mutagenesis kit (Agilent Technologies, Santa Clara, CA, USA). The reporter constructs containing the 3 antioxidant response element (3xARE) promoter in pGL vector were kindly donated by Dr. Raymond J Deshaies. The reporter construct for the *MMP9* promoter was donated by Dr. Thomas Iftner. Reporter assays using these clones were conducted using the DualGlo-Luciferase Reporter Assay System (Promega, Madison, WI, USA) according to the manufacturer’s recommended protocols. Control vectors, pCDNA, and FLAG CMV were also used for control experiments. Specific primers were designed for mutagenesis using the QuikChange Primer Design tool (Agilent Technologies, Santa Clara, CA, USA). Mutated sequences of the DLG motif were confirmed using Sanger sequencing (Genomed, Warsaw, Poland). The primer sequences used for the mutagenesis of the DLG motif were purchased from Genomed (Warsaw, Poland). The primer sequences used for the mutagenesis of the DLG motif were purchased from Genomed (Warsaw, Poland) and are depicted in the following Table 2. Permission for the facility to perform experiments with microorganisms and genetically modified organisms was provided by Minister of the Environment, Poland (Decision number 132/2016). 

### 4.3. Cell Transfections and Luciferase Assays

To validate the transcriptional activity of NRF2 (WT and MTs), a dual luciferase assay was performed. Hepa1-6 and Huh7 cells (2 × 10^4^ cells/well) were grown to 40–70% confluency in 24-well plates and transiently co-transfected with the reporter and effector plasmids (that are indicated in Figure legends) with 100 ng of TK-LUC renilla plasmid as an internal control using Lipofectamine 3000 (Thermo Fisher Scientific, Waltham, MA, USA), according to the manufacturer’s instructions. Depending on experimental design, we transfected different plasmids accordingly. For KEAP1 co-transfections, 50 ng of KEAP1 plasmid/well was used. Cells were harvested after 48 h and luciferase activity was assayed using a Luciferase Assay Kit (Promega, Madison, WI, USA). Firefly luciferase activity was normalized with Renilla luciferase to control for sample-to-sample variations in transfection efficiency. All reporter assays were repeated independently at least 3 times. Luminescence was measured using a Synergy LX luminometer (Biotek, Winooski, VT, USA).

### 4.4. Statistical Analysis

Data are presented as the mean ± standard error of the mean (SEM) of each group in the experiment. The statistical analysis was done using a one-way analysis of variance (ANOVA), followed by Tukey’s post hoc tests. Any *p*-value < 0.05 was considered statistically significant. GraphPad PRISM software version 6 (GraphPad Software, La Jolla, CA, USA) was used for the statistical analysis.

## Figures and Tables

**Table 1:** Novel human NRF2 DLG MTs identified in different cancers and in ICGC database.

MT ID	DNA Change	Type	Amino Acid Change	Project	Tumor Type	Tumor Subtype	Donors Affected
MU1324215	chr2:g.178098960C>G	single base substitution	D29H	LUSC-US	Lung cancer	Squamous cell carcinoma	5/485 (1.03%)
				CESC-US	Cervical cancer	Cervical squamous cell carcinoma	2/289 (0.69%)
				HNSC-US	Head and neck cancer	Squamous cell carcinoma	3/508 (0.59%)
				LUSC-KR	Lung cancer	Adenocarcinoma, squamous cell carcinoma	1/170 (0.59%)
				BLCA-US	Bladder cancer	Invasive urothelial bladder cancer	2/411 (0.49%)
				LICA-FR	Liver cancer	Hepatocellular carcinoma (secondary to alcohol and adiposity)	1/252 (0.40%)
				ESCA-CN	Esophageal cancer	Squamous carcinoma	1/332 (0.30%)
				UCEC-US	Endometrial cancer	Uterine corpus endometrial carcinoma	1/531 (0.19%)
				LUAD-US	Lung cancer	Adenocarcinoma	1/516 (0.19%)
MU1327674	chr2:g.178098960C>T	single base substitution	D29N	LUSC-US	Lung cancer	Squamous cell carcinoma	5/485 (1.03%)
				LUSC-KR	Lung cancer	Adenocarcinoma, squamous cell carcinoma	1/170 (0.59%)
				LICA-CN	Liver cancer	Hepatocellular carcinoma HBV-associated	1/402 (0.25%)
				LINC-JP	Liver cancer	Hepatocellular carcinoma (virus associated)	1/394 (0.25%)
MU1316143	chr2:g.178098960C>A	single base substitution	D29Y	LUSC-US	Lung cancer	Squamous cell carcinoma	2/485 (0.41%)
				CESC-US	Cervical cancer	Cervical squamous cell carcinoma	1/289 (0.35%)
				BLCA-US	Bladder cancer	Invasive urothelial bladder cancer	1/411 (0.24%)
				HNSC-US	Head and Neck cancer	Squamous cell carcinoma	1/508 (0.20%)
MU871836	chr2:g.178098959T>C	single base substitution	D29G	LICA-FR	Liver cancer	Hepatocellular carcinoma (secondary to alcohol and adiposity)	2/252 (0.79%)
				LUSC-KR	Lung cancer	Adenocarcinoma, squamous cell carcinoma	1/170 (0.59%)
				ORCA-IN	Oral cancer	Gingivobuccal	1/178 (0.56%)
				LINC-JP	Liver cancer	Hepatocellular carcinoma (virus associated)	2/394 (0.51%)
				LUSC-US	Lung cancer	Squamous cell carcinoma	2/485 (0.41%)
				LICA-CN	Liver cancer	Hepatocellular carcinoma HBV-associated	1/402 (0.25%)
				LUAD-US	Lung cancer	Adenocarcinoma	1/516 (0.19%)
MU1330977	chr2:g.178098957G>A	single base substitution	L30F	LUSC-US	Lung cancer	Squamous cell carcinoma	4/485 (0.82%)
				LIRI-JP	Liver cancer	Hepatocellular carcinoma (virus associated)	1/258 (0.39%)
				PACA-CA	Pancreatic cancer	Ductal adenocarcinoma	1/268 (0.37%)
				HNSC-US	Head and neck cancer	Squamous cell carcinoma	1/508 (0.20%)
MU1292484	chr2:g.178098953C>G	single base substitution	G31A	LUSC-US	Lung cancer	Squamous cell carcinoma	5/485 (1.03%)
				ESCA-CN	Esophageal cancer	Squamous carcinoma	1/332 (0.30%)
				BLCA-US	Bladder cancer	Invasive urothelial bladder cancer	1/411 (0.24%)
MU866686	chr2:g.178098953C>T	single base substitution	G31E	LUSC-KR	Lung cancer	Adenocarcinoma, squamous cell carcinoma	2/170 (1.18%)
				LINC-JP	Liver cancer	Hepatocellular carcinoma (Virus associated)	2/394 (0.51%)
MU83818151	chr2:g.178098954C>T	single base substitution	G31R	LICA-FR	Liver cancer	Hepatocellular carcinoma (secondary to alcohol and adiposity)	1/252 (0.40%)
				BLCA-US	Bladder cancer	Invasive urothelial bladder cancer	1/411 (0.24%)
				LUAD-US	Lung cancer	Adenocarcinoma	1/516 (0.19%)
MU623518	chr2:g.178098956A>T	single base substitution	L30H	KIRC-US	Renal cancer	Clear cell carcinoma	1/361 (0.28%)
				HNSC-US	Head and neck cancer	Squamous cell carcinoma	1/508 (0.20%)
MU130685128	chr2:g.178098953C>A	single base substitution	G31V	LUSC-US	Lung cancer	Squamous cell carcinoma	1/485 (0.21%)
MU830878	chr2:g.178098956A>C	single base substitution	L30R	LINC-JP	Liver cancer	Hepatocellular carcinoma (virus associated)	3/394 (0.76%)
				KIRP-US	Renal cancer	Papillary carcinoma	1/278 (0.36%)
MU131168581	chr2:g.178098956A>G	single base substitution	L30P	HNSC-US	Head and neck cancer	Squamous cell carcinoma	1/508 (0.20%)
MU29615597	chr2:g.178098959T>G	single base substitution	D29A	LIRI-JP	Liver cancer	Hepatocellular carcinoma (virus associated)	1/258 (0.39%)

**Table 2:** Primer pairs used in our study.

Primer Name	Primer Sequence	Species
D29A	F: CTCGACTTACTCCAAGAGCTATATCTTGCCTCCAAAGTA R: TACTTTGGAGGCAAGATATAGCTCTTGGAGTAAGTCGAG	Human
L30F	F: CTCGACTTACTCCAAAATCTATATCTTGCCTCCAAAGTATGTCA R: TGACATACTTTGGAGGCAAGATATAGATTTTGGAGTAAGTCGAG	Human
D29A	F: CTCGACTTACTCCAAGAGCTATGTCTTGCCTCCAA R: TTGGAGGCAAGACATAGCTCTTGGAGTAAGTCGAG	Mouse
L30F	F: CGACTTACTCCAAAATCTATGTCTTGCCTCCAAAGGAT R: ATCCTTTGGAGGCAAGACATAGATTTTGGAGTAAGTCG	Mouse

## Abbreviations

ARE	Antioxidant response element
BRAF	V-raf murine sarcoma viral oncogene homolog B1
CMD	Choline- devoid methionine-deficient
CUL3 E3	Cullin 3-RING E3
DEN	Diethylnitrosamine
DMEM	Dulbecco’s modified eagle medium
ERK	Extracellular signal-regulated kinases
ESCA	Esophageal carcinoma
FBS	Fetal bovine serum
Hepa 1-6	Mouse hepatoma cell
HBV	Hepatitis B virus
HCC	Hepatocellular carcinoma
HCV	Hepatitis C virus
HepG2	Human hepatoma cell line
Huh-7	Human hepatoma cells-7
ICGC	International Cancer Genome Consortium
JUN	Putative transforming gene of avian sarcoma virus 17
KEAP1	Kelch-like ECH-associated protein 1
KO	Knockout
K-Ras	Kirsten rat sarcoma viral oncogene homolog
LUSC	Lung squamous cell carcinoma
MAPK	Mitogen-activated protein kinase
MEK	MAPK or ERK kinases
MMP-9	Matrix metalloproteinase 9
MYC	Cellular homolog of the retroviral v-Myc oncogene
NAFLD	Non-alcoholic fatty liver disease
NASH	Non-alcoholic steatohepatitis
NSCLC	Non-small cell lung cancer
NRF2	NF-E2-related factor 2
UCEC	Uterine corpus endometrial carcinoma

## References

[1] Lin L., Yan L., Liu Y., Qu C., Ni J., Li H. (2020). The Burden and Trends of Primary Liver Cancer Caused by Specific Etiologies from 1990 to 2017 at the Global, Regional, National, Age, and Sex Level Results from the Global Burden of Disease Study 2017. Liver Cancer.

[2] De Martel C., Maucort-Boulch D., Plummer M., Franceschi S. (2015). World-wide relative contribution of hepatitis B and C viruses in hepatocellular carcinoma. Hepatology.

[3] Kanda T., Goto T., Hirotsu Y., Masuzaki R., Moriyama M., Omata M. (2020). Molecular Mechanisms: Connections between Nonalcoholic Fatty Liver Disease, Steatohepatitis and Hepatocellular Carcinoma. Int. J. Mol. Sci..

[4] Michelotti G.A., Machado M.V., Diehl A.M. (2013). NAFLD, NASH and liver cancer. Nat. Rev. Gastroenterol. Hepatol..

[5] Sidharthan S., Kottilil S. (2014). Mechanisms of alcohol-induced hepatocellular carcinoma. Hepatol. Int..

[6] White D.L., Kanwal F., El-Serag H.B. (2012). Association between nonalcoholic fatty liver disease and risk for hepatocellular cancer, based on systematic review. Clin. Gastroenterol. Hepatol..

[7] Yang H.I., Lu S.N., Liaw Y.F., You S.L., Sun C.A., Wang L.Y., Hsiao C.K., Chen P.J., Chen D.S., Chen C.J. (2002). Hepatitis B e antigen and the risk of hepatocellular carcinoma. N. Engl. J. Med..

[8] Yang J.D., Kim W.R., Coelho R., Mettler T.A., Benson J.T., Sanderson S.O., Therneau T.M., Kim B., Roberts L.R. (2011). Cirrhosis is present in most patients with hepatitis B and hepatocellular carcinoma. Clin. Gastroenterol. Hepatol..

[9] Ramezani A., Nahad M.P., Faghihloo E. (2018). The role of Nrf2 transcription factor in viral infection. J. Cell. Biochem..

[10] Ngo H.K.C., Kim D.H., Cha Y.N., Na H.K., Surh Y.J. (2017). Nrf2 Mutagenic Activation Drives Hepatocarcinogenesis. Cancer Res..

[11] Zhang M., Zhang C., Zhang L., Yang Q., Zhou S., Wen Q., Wang J. (2015). Nrf2 is a potential prognostic marker and promotes proliferation and invasion in human hepatocellular carcinoma. BMC Cancer.

[12] Shibata T., Ohta T., Tong K.I., Kokubu A., Odogawa R., Tsuta K., Asamura H., Yamamoto M., Hirohashi S. (2008). Cancer related mutations in NRF2 impair its recognition by Keap1-Cul3 E3 ligase and promote malignancy. Proc. Natl. Acad. Sci. USA.

[13] Guichard C., Amaddeo G., Imbeaud S., Ladeiro Y., Pelletier L., Maad I.B., Calderaro J., Bioulac-Sage P., Letexier M., Degos F. (2012). Integrated analysis of somatic mutations and focal copy-number changes identifies key genes and pathways in hepatocellular carcinoma. Nat. Genet..

[14] Fukutomi T., Takagi K., Mizushima T., Ohuchi N., Yamamoto M. (2014). Kinetic, thermodynamic, and structural characterizations of the association between Nrf2-DLGex degron and Keap1. Mol. Cell. Biol..

[15] Orrù C., Perra A., Kowalik M.A., Rizzolio S., Puliga E., Cabras L., Giordano S., Columbano A. (2020). Distinct Mechanisms Are Responsible for Nrf2-Keap1 Pathway Activation at Different Stages of Rat Hepatocarcinogenesis. Cancers (Basel).

[16] Shibata T., Kokubu A., Saito S., Narisawa-Saito M., Sasaki H., Aoyagi K., Yoshimatsu Y., Tachimori Y., Kushima R., Kiyono T. (2011). NRF2 mutation confers malignant potential and resistance to chemoradiation therapy in advanced esophageal squamous cancer. Neoplasia.

[17] Ohta T., Iijima K., Miyamoto M., Nakahara I., Tanaka H., Ohtsuji M., Suzuki T., Kobayashi A., Yokota J., Sakiyama T. (2008). Loss of Keap1 function activates Nrf2 and provides advantages for lung cancer cell growth. Cancer Res..

[18] Endo H., Owada S., Inagaki Y., Shida Y., Tatemichi M. (2018). Glucose starvation induces LKB1-AMPK-mediated MMP-9 expression in cancer cells. Sci. Rep..

[19] Chen H.H., Chang H.H., Chang J.Y., Tang Y.C., Cheng Y.C., Lin L.M., Cheng S.Y., Huang C.H., Sun M.W., Chen C.T. (2017). Enhanced B-Raf-mediated NRF2 gene transcription and HATs-mediated NRF2 protein acetylation contributes to ABCC1-mediated chemoresistance and glutathione-mediated survival in acquired topoisomerase II poison-resistant cancer cells. Free Radic. Biol. Med..

[20] Maurer G., Tarkowski B., Baccarini M. (2011). Raf kinases in cancer-roles and therapeutic opportunities. Oncogene.

[21] DeNicola G.M., Karreth F.A., Humpton T.J., Gopinathan A., Wei C., Frese K., Mangal D., Yu K.H., Yeo C.J., Calhoun E.S. (2011). Oncogene-induced Nrf2 transcription promotes ROS detoxification and tumorigenesis. Nature.

[22] Pratilas C.A., Taylor B.S., Ye Q., Viale A., Sander C., Solit D.B., Rosen N. (2009). (V600E)BRAF is associated with disabled feedback inhibition of RAF-MEK signaling and elevated transcriptional output of the pathway. Proc. Natl. Acad. Sci. USA.

[23] Ritt D.A., Monson D.M., Specht S.I., Morrison D.K. (2010). Impact of feedback phosphorylation and Raf heterodimerization on normal and mutant B-Raf signaling. Mol. Cell. Biol..

[24] Mitsuishi Y., Motohashi H., Yamamoto M. (2012). The Keap1-Nrf2 system in cancers: Stress response and anabolic metabolism. Front. Oncol..

[25] Salemi R., Falzone L., Madonna G., Polesel J., Cinà D., Mallardo D., Ascierto P.A., Libra M., Candido S. (2018). MMP-9 as a Candidate Marker of Response to BRAF Inhibitors in Melanoma Patients With BRAF(V600E) Mutation Detected in Circulating-Free DNA. Front. Pharmacol..

[26] Khamari R., Trinh A., Gabert P.E., Corazao-Rozas P., Riveros-Cruz S., Balayssac S., Malet-Martino M., Dekiouk S., Joncquel Chevalier Curt M., Maboudou P. (2018). Glucose metabolism and NRF2 coordinate the antioxidant response in melanoma resistant to MAPK inhibitors. Cell Death Dis..

[27] Zhang J., Bajari R., Andric D., Gerthoffert F., Lepsa A., Nahal-Bose H., Stein L.D., Ferretti V. (2019). The International Cancer Genome Consortium Data Portal. Nat. Biotechnol..

[28] Pan H., Wang H., Zhu L., Mao L., Qiao L., Su X. (2013). The role of Nrf2 in migration and invasion of human glioma cell U251. World Neurosurg..

[29] Frasca F., Nucera C., Pellegriti G., Gangemi P., Attard M., Stella M., Loda M., Vella V., Giordano C., Trimarchi F. (2008). BRAF(V600E) mutation and the biology of papillary thyroid cancer. Endocr. Relat. Cancer.

[30] Guarneri C., Bevelacqua V., Polesel J., Falzone L., Cannavò P.S., Spandidos D.A., Malaponte G., Libra M. (2017). NF-κB inhibition is associated with OPN/MMP-9 downregulation in cutaneous melanoma. Oncol. Rep..

[31] Chu X.Y., Li Z.J., Zheng Z.W., Tao Y.L., Zou F.X., Yang X.F. (2018). KEAP1/NRF2 signaling pathway mutations in cervical cancer. Eur. Rev. Med. Pharmacol. Sci..

[32] Kerins M.J., Ooi A. (2018). A catalogue of somatic NRF2 gain-of-function mutations in cancer. Sci. Rep..

[33] Zhang M., Zhang L., Li Y., Sun F., Fang Y., Zhang R., Wu J., Zhou G., Song H., Xue L. (2020). Exome sequencing identifies somatic mutations in novel driver genes in non-small cell lung cancer. Aging.

[34] McMahon M., Thomas N., Itoh K., Yamamoto M., Hayes J.D. (2004). Redox-regulated turnover of Nrf2 is determined by at least two separate protein domains, the redox-sensitive Neh2 degron and the redox-insensitive Neh6 degron. J. Biol. Chem..

[35] Tong K.I., Katoh Y., Kusunoki H., Itoh K., Tanaka T., Yamamoto M. (2006). Keap1 recruits Neh2 through binding to ETGE and DLG motifs: Characterization of the two-site molecular recognition model. Mol. Cell. Biol..

[36] Tong K.I., Padmanabhan B., Kobayashi A., Shang C., Hirotsu Y., Yokoyama S., Yamamoto M. (2007). Different electrostatic potentials define ETGE and DLG motifs as hinge and latch in oxidative stress response. Mol. Cell. Biol..

[37] Orrù C., Szydlowska M., Taguchi K., Zavattari P., Perra A., Yamamoto M., Columbano A. (2018). Genetic inactivation of Nrf2 prevents clonal expansion of initiated cells in a nutritional model of rat hepatocarcinogenesis. J. Hepatol..

[38] Ziv E., Zhang Y., Kelly L., Nikolovski I., Boas F.E., Erinjeri J.P., Cai L., Petre E.N., Brody L.A., Covey A.M. (2020). NRF2 Dysregulation in Hepatocellular Carcinoma and Ischemia: A Cohort Study and Laboratory Investigation. Radiology.

[39] Fujimoto A., Furuta M., Totoki Y., Tsunoda T., Kato M., Shiraishi Y., Tanaka H., Taniguchi H., Kawakami Y., Ueno M. (2016). Whole-genome mutational landscape and characterization of noncoding and structural mutations in liver cancer. Nat. Genet..

[40] Inami Y., Waguri S., Sakamoto A., Kouno T., Nakada K., Hino O., Watanabe S., Ando J., Iwadate M., Yamamoto M. (2011). Persistent activation of Nrf2 through p62 in hepatocellular carcinoma cells. J. Cell Biol..

[41] Zavattari P., Perra A., Menegon S., Kowalik M.A., Petrelli A., Angioni M.M., Follenzi A., Quagliata L., Ledda-Columbano G.M., Terracciano L. (2015). Nrf2, but not β-catenin, mutation represents an early event in rat hepatocarcinogenesis. Hepatology.

[42] Kobayashi A., Kang M.I., Okawa H., Ohtsuji M., Zenke Y., Chiba T., Igarashi K., Yamamoto M. (2004). Oxidative stress sensor Keap1 functions as an adaptor for Cul3-based E3 ligase to regulate proteasomal degradation of Nrf2. Mol. Cell Biol..

[43] Gong M., Li Y., Ye X., Zhang L., Wang Z., Xu X., Shen Y., Zheng C. (2020). Loss-of-function mutations in KEAP1 drive lung cancer progression via KEAP1/NRF2 pathway activation. Cell Commun. Signal..

[44] Bergers G., Brekken R., McMahon G., Vu T.H., Itoh T., Tamaki K., Tanzawa K., Thorpe P., Itohara S., Werb Z. (2000). Matrix metalloproteinase-9 triggers the angiogenic switch during carcinogenesis. Nat. Cell Biol..

[45] Zipper L.M., Mulcahy R.T. (2003). Erk activation is required for Nrf2 nuclear localization during pyrrolidine dithiocarbamate induction of glutamate cysteine ligase modulatory gene expression in HepG2 cells. Toxicol. Sci..

